# Parametric response map (prm) is a promising tool for the monitoring of post traumatic cerebral oedema

**DOI:** 10.1186/2197-425X-3-S1-A440

**Published:** 2015-10-01

**Authors:** J Grèze, B Lemasson, E Barbier, J-F Payen, P Bouzat

**Affiliations:** CHU Grenoble, Anesthesia-Intensive Care, Grenoble, France; University of Grenoble, Grenoble Institute of Neuroscience, Grenoble, France

## Introduction

Cerebral edema (CE) is the key factor for the development of secondary brain injuries after severe traumatic brain injury (TBI). This edema may be assessed using the averaged Apparent diffusion coefficient (ADC) measured by diffusion-weighted MRI in specified ROI. However, this approach does not take into account the complexity and the heterogeneity of the post traumatic edema. The parametric response map (PRM), a voxel-based analysis technique, is a promising tool to better investigate spatially dispersed changes of ADC over time (1).

## Objectives

In this study, we compared the PRM analysis to the classical ADCaveraged approach to characterize evolution of the ADC in the hours following a rat TBI.

## Methods

Eighteen male Wistar rats were traumatized (TBI group) and 7 sham operated rats were used for the control group. Injury was induced using the impact-acceleration protocol (2). Diffusion-weighted images were acquired using an echo-planar, spin-echo, sequence. Acquisitions were performed before the trauma (H.ref), immediately after (H0), 60 min after (H1) and 120 min (H2) after the trauma. Two ROI were contoured, one including the cortex (Cortical ROI) and the other including most of the brain (Brain ROI). For each rat, each time point and ROI, PRM was used to analyze, voxel-wise, changes in ADC. PRM was performed in specified ROI by calculating the difference in ADC values of each voxel between H.ref and the values of the other time points. A variation threshold (100 µm²/s) was defined as the variation value below which 95% of the voxels in the sham rats were considered as stable by the PRM analysis. We computed then the fraction of voxels whose ADC significantly increased (PRM+: red), the fraction of voxels whose ADC significantly decreased (PRM-: blue), and the fraction of voxels whose ADC was unchanged (PRM0: green). In addition, mean ADC inside the ROI was computed.

## Results

Classical approach showed a difference between TBI group and Sham group in Cortical ROI ( p = 0.04) but not in Brain ROI (p = 0.32). PRM approach on the Brain ROI detected edematous processes: TBI group presented statistically fewer PRM0 voxels than Sham group at every time point (p < 0.05), with a dual increase of red and blue voxels. We sorted traumatized rats based on their mean ADC at H2 in vasogenic (increased ADC) or cytotoxic (decreased ADC) subgroups, and PRM analysis performed immediately after TBI was able to precisely predict the type of edema at H2 (specificity 100%, sensibility 88.7%).

## Conclusions

PRM analysis is a valuable tool for analyzing the complexity of post traumatic CE. PRM was able to detect CE when classical averaged approach failed. Our data showed that a single model of experimental TBI induced several types of CE evolutions. PRM could identify specific subgroups for clinical practice or for future experimental trials.

## Grant Acknowledgment

'Fondation Gueules Cassées'
